# Allogenic hematopoietic stem cell transplantation outcomes of patients aged ≥ 55 years with acute myeloid leukemia or myelodysplastic syndromes in China: a retrospective study

**DOI:** 10.1186/s13287-024-03640-4

**Published:** 2024-01-29

**Authors:** Lu Gao, Li Yang, Shiyuan Zhou, Wenjuan Zhu, Yue Han, Suning Chen, Shengli Xue, Ying Wang, Huiying Qiu, Depei Wu, Xiaojin Wu

**Affiliations:** 1 Department of Hematology, The First Affiliated Hospital of Soochow University, Suzhou, China; 2 National Clinical Research Center for Hematologic Diseases, Jiangsu Institute of Hematology, Suzhou, China; 3 Institute of Blood and Marrow Transplantation, Collaborative Innovation Center of Hematology, Soochow University, Suzhou, China; 4 Tongren Hospital Shanghai Jiao Tong University School of Medicine, 111 Xianxia Road, Shanghai, China

**Keywords:** Old patients, Refractory or relapsed, Allogenic hematopoietic stem cell transplantation, AML, MDS

## Abstract

**Background:**

Elderly patients with acute myeloid leukemia or myelodysplastic syndromes (AML/MDS) have historically had poor prognoses. However, there has been a recent increase in the use of allogenic hematopoietic stem cell transplantation (allo-HSCT) are in this patient population. Nevertheless, the optimal choice of donor type for the patients remains an unmet need. Limited data exist on the use of allo-HSCT in elderly patients with AML/MDS from China. To better understand and optimize the selection of donor type for the elderly patients, particularly for those with refractory or relapsed disease, in comparison with the previous studies in the US and Europe.

**Methods:**

Our retrospective study enrolled 259 patients aged over 55 years who underwent their first allo-HSCT between April 2015 and August 2022. These patients were divided into three groups based on donor type: haploidentical related donor group (haploidentical related donor transplantation [HID], n = 184), matched sibling donor group (matched sibling donor transplantation [MSD], n = 39), and matched unrelated donor group (matched unrelated donor transplantation [MUD], n = 36). Statistics were performed with the chi-square test, the log-rank and Fine-Gray tests.

**Results:**

The median age of the cohort was 57 years (range: 55–75) and 26.25% of patients were over 60 years old. Younger patients had a higher incidence of acute graft-versus-host disease (HR = 1.942, P = 0.035), faster neutrophil recovery (HR = 1.387, P = 0.012), and better overall survival (HR = 0.567, P = 0.043) than patients aged ≥ 60 years across the entire cohort. Patients with refractory or relapsed (R/R) diseases had delayed neutrophil engraftment (P = 0.010, HR = 0.752) and platelet engraftment (P < 0.001, HR = 0.596), higher incidence of relapses (HR = 2.300, P = 0.013), and inferior relapse-free survival (RFS) (HR = 1.740, HR = 0.016) regardless of donor type. When it came to graft-versus-host-disease-free, relapse-free survival (GRFS), MUDs turned out to be superior to HIDs (HR = 0.472, P = 0.026) according to the multivariable analysis. In contrast, we found MSDs had an inferior GRFS to HIDs in parallel (HR = 1.621, P = 0.043).

**Conclusion:**

The choice of donor type did not significantly affect the outcomes of allo-HSCT. However, when considering the quality of post-transplant life, MUDs or HIDs from younger donors may be the optimal choice for elderly patients.

**Supplementary Information:**

The online version contains supplementary material available at 10.1186/s13287-024-03640-4.

## Introduction

Acute myeloid leukemia (AML) and myelodysplastic syndromes (MDSs) are the most prevalent malignant hematologic disorders among older adults [1]. While the decision to use allogenic hematopoietic stem cell transplantation (allo-HSCT) in geriatric patients with AML or MDS remains cautious due to aging issues, allo-HSCT remains the only potentially curative treatment for these patients. Refractory or relapsed (R/R) disease status in elderly patients with AML/MDS adds to the challenge of allo-HSCT. With an expanded older population, increased transplantation needs can be met partly through the development of reduced transplant-related toxicity as the myeloablative regimens remain the first choice in allo-HSCT. Meanwhile, the probability of finding a matched sibling donor (MSD) decreased with age after 55 [2], and matched sibling donors are conventionally recommended as the first-line choice in HSCT. In addition to MSDs, matched unrelated donors (MUDs) provided by national marrow donor programs or institutions can be another option. However, the prolonged search procedure for MUDs may result in treatment delays, during which clinical deterioration along with physical decay can disqualify patients from allo-HSCT. Therefore, haploidentical related donor (HID)0 transplantation, which features rapid and adequate donors, has become the third well-developed transplantation donor source for patients aged ≥ 55 years in the near term [3].

To date, several studies have shown that allo-HSCT from HID, MSD, and MUD have similar overall survival (OS) and relapse-free survival (RFS) rates [4–10]. This suggests that HID could be a valid alternative for elderly patients who do not have a matched donor available and have been diagnosed with refractory or relapsed (R/R) diseases. However, another study has demonstrated that increasing donor age is associated with worse OS, along with other transplant-related complications [11]. Given the limited research related to the uncertain situation among patients aged over 55 years old, we conducted a retrospective comparison in a real-world setting between HID from offspring donors and MSD from sibling donors or MUD from younger donors (≤ 35).

## Patients and methods

### Study design

This retrospective real-world analysis enrolled consecutive patients aged ≥ 55 years with a diagnosis of AML or MDS who underwent their first allo-HSCT between April 2015 and August 2022, based on data from the First Affiliated Hospital of Soochow University. Informed consent was obtained in accordance with the Declaration of Helsinki and approved by the Faculty Hospital Ethics Committee at the First Affiliated Hospital of Soochow University in China. The 259 patients in the study were separated into three groups based on their donor selection: haploidentical related donor (5–8/10 HLA matching assessing HLA-A, B, C, DR, and DQ loci) from their offspring (184 patients included), HLA-matched sibling donor (39 patients included), and HLA-matched unrelated donor (9 or 10/10 HLA matching) (36 patients included). Parameters were prospectively collected before transplantations, including age (< 60 or ≥ 60), sex, Eastern Cooperative Oncology Group Zubrod performance status (ECOG), the hematopoietic cell transplantation comorbidity index (HCT-CI) [12], disease status before stem cell transplantation (the first complete remission [CR1]or non-CR1), European Leukemia Net guideline-based molecular risk for AML and Revised International Prognostic Scoring System for MDS [13, 14], transplant conditioning intensity score (TCI, < 4.0 or ≥ 4.0), graft cell source, refractory or relapsed, donor-to-recipient blood type matching, and gender matching. The primary endpoints were OS, transplant-related mortality (TRM), RFS, and graft-versus-host-disease-free, relapse-free survival (GRFS). The secondary endpoints were successful engraftment of platelets and neutrophils, incidences of acute graft-versus-host disease (GvHD), chronic GvHD, Cytomegalovirus (CMV), and Epstein–Barr virus (EBV). Endpoints were measured from the first date of allo-HSCT to the date of event or last follow-up.

### Treatment

Patients were treated with following standard transplant protocols: either a modified Busulfan and Cyclophosphamide (Bu/Cy) conditioning regimen including cytosine arabinoside 2 g/m^2^/12 h (on days − 10 and − 9), Busulfan 0.8 mg/kg/6 h (from days − 8 to − 6), and cyclophosphamide 1.8 g/m^2^/day (on days − 4 and − 3), or a reduced-intensity preparative regimen that consisted of Fludarabine 30 mg/m^2^/day (from days − 7 to − 4), cytosine arabinoside 2 g/m^2^/day (on day − 7), Busulfan 3.2 mg/kg/day (from days − 6 to − 5), Melphalan 100 mg/m^2^/day (from days − 4 to − 3), or Fludarabine 30 mg/m^2^/day (from days − 9 to − 5), cytosine arabinoside 1.5 g/m^2^/day (on days from − 9 to − 5), and Busulfan 3.2 mg/kg (on days − 4 and − 3). The conditioning regimens were evaluated with TCI [15]. The Bu/Cy conditioning regimen belonged to high intensity group (TCI ≥ 4) while the fludarabine-based conditioning regimen was classified into reduced-intensity group (TCI < 4). GvHD prophylaxis was administered to those accepting stem cells from an HLA-haploidentical or matched unrelated donor, and it was composed of cyclophosphamide, short-range methotrexate, mycophenolate mofetil, and rabbit anti-thymocyte globin. For those accepting stem cells from HLA-matched related donors, only cyclosporine was administered. To eliminate the positive antibody against human leukocyte antigen detected in patients, CD20 antibody or plasma exchange was applied before transplantation.

### Definition and statistical analysis

Categorical variables at baseline were presented as percentages and continuous variables were presented as mean or median values. Patient characteristics were compared among different donor types using the Kruskal–Wallis test for continuous variables and the chi-square test or Fisher’s exact test for categorical or hierarchical features. OS, RFS, and GRFS were estimated using the Kaplan–Meier method. Cumulative incidences of transplant-related mortality, engraftment, GvHD, and infection were calculated considering competing risks (death and relapse for chronic GvHD). Engraftment was defined as an absolute neutrophil count greater than 500 (> 0.5 × 10^9^/L) on the first day of three consecutive days. Platelet recovery was defined as a platelet count greater than 20 × 10^9^/L on the first day of seven consecutive days without platelet transfusion [16]. EBV and CMV activation were defined as DNA viral load monitoring points with more than 100 copies/mL [17]. GRFS was defined as the first occurrence among grade III–IV acute GVHD, extensive chronic GVHD, relapse, or death [18]. Univariate analysis was performed using the log-rank and Fine-Gray tests, accounting for potential prognostic parameters. Cox proportional hazards regression models were used to weigh and adjust risk factors (variables with P < 0.100 were included) with the main interest variable anchored to donor type. Backward elimination was applied for the final models. Statistical significance was set at P < 0.050 for two-sided tests. Statistical analyses were performed with the SPSS 25.0 and R 4.3.0 software packages.

## Results

### Patient and donor characteristics

The 259 patients were included in the study and divided into three groups based on donor type: HID (n = 184), MSD (n = 39), and MUD (n = 36). The median age of the entire cohort was 57 years (ranging from 55 to 75), with 68 patients (26%) over 60 years old. Of all patients, 64% of patients (167 patients) were diagnosed with AML, while 111 patients (43%) had refractory or relapsed diseases before receiving allo-HSCT. Among the 68 patients aged over 60 years old, 31 (45%, P = 0.596) had a diagnosis of R/RAML/MDS, which was significantly higher than the percentage in the younger cohort (25%, P < 0.001). Table 1 provides more detailed information on the study.

**Table 1:** Patients and transplantation characteristics

Characteristics	HID	MSD	MUD	P-value
	N = 184	N = 39	N = 36	
*Age*				0.406
Below sixty	132 (72%)	32 (82%)	27 (75%)	
Over sixty	52 (28%)	7 (18%)	9 (25%)	
*Sex*				0.503
Female	59 (32%)	12 (31%)	15 (42%)	
Male	125 (68%)	27 (69%)	21 (58%)	
*Diagnosis*				0.089
AML	126 (68%)	20 (51%)	21 (58%)	
MDS	58 (31%)	19 (49%)	15 (42%)	
*ECOG*				0.744
0–1	150 (81%)	33 (85%)	30 (83%)	
2	27 (15%)	4 (10%)	6 (17%)	
3–4	7 (4%)	2 (5%)	0 (0%)	
*HCT-CI*				0.778
0–1	125 (68%)	27 (69%)	27 (75%)	
2–3	48 (26%)	10 (26%)	6 (17%)	
4–6	11 (6%)	2 (5%)	3 (8%)	
*TCI*				0.481
< 4	59 (32%)	11 (28%)	8 (22%)	
≥ 4	125 (68%)	28 (72%)	28 (78%)	
*Refractory or relapsed*				0.360
No	100 (54%)	25 (64%)	23 (64%)	
Yes	84 (46%)	14 (36%)	13 (36%)	
*Cytogenetic evaluation*^†^				0.162
Favourable or intermediate risk	117 (64%)	22 (56%)	17 (47%)	
Adverse risk	67 (36%)	17 (44%)	19 (53%)	
*CR1*				0.451
Not	110 (60%)	26 (67%)	25 (69%)	
Yes	74 (40%)	13 (33%)	11 (31%)	
*Donor to recipient*				0.017*
Others	144 (78%)	34 (87%)	35 (97%)	
Female to male	40 (22%)	5 (13%)	1 (3%)	
*Anti-HLA*				0.683
Negative	157 (85%)	34 (87%)	33 (92%)	
Positive	27 (15%)	5 (13%)	3 (8%)	
*Graft source*				< 0.001*
PB	104 (56%)	30 (77%)	35 (97%)	
BM	5 (3%)	0 (0%)	0 (0%)	
PB + BM	75 (41%)	9 (23%)	1 (3%)	
*ABO blood type*				0.026*
Matched	89 (48%)	26 (67%)	13 (36%)	
Not matched	95 (52%)	13 (33%)	23 (64%)	
MNC (median)	10.5	10.2	8.3	0.010*
CD34+ cells(median)	1.9	2.0	1.4	< 0.001*

*HCT-CI* the hematopoietic cell transplantation comorbidity index, *ECOG* Zubrod-ECOG-WHO from Eastern Cooperative Oncology Group, *TCI* transplant conditioning intensity score, *HLA* human leukocyte antigen, *CR1* the first complete remission, *MNC* mononuclear cell

^†^Cytogenetic criteria definition: adverse: − 7, inv(3)/t(3q)/del(3q), double including -7/del(7q), or complex (3 abnormalities), complex > 3 abnormalities for MDS; t(6;9)(p23;q34.1)/DEK::NUP214, t(v;11q23.3)/KMT2A-rearranged, t(9;22)(q34.1;q11.2)/BCR::ABL1, t(8;16)(p11;p13)/KAT6A::CREBBP, inv(3)(q21.3q26.2) or t(3;3)(q21.3;q26.2)/GATA2, MECOM(EVI1), t(3q26.2;v)/MECOM(EVI1)-rearranged, − 5 or del(5q); − 7; − 17/abn(17p), Complex karyotype, monosomal karyotype, Mutated ASXL1, BCOR, EZH2, RUNX1, SF3B1, SRSF2, STAG2, U2AF1, or ZRSR2, Mutated TP53 for AML

*Significant at P < 0.05

### Engraftment

**Fig. 1:**
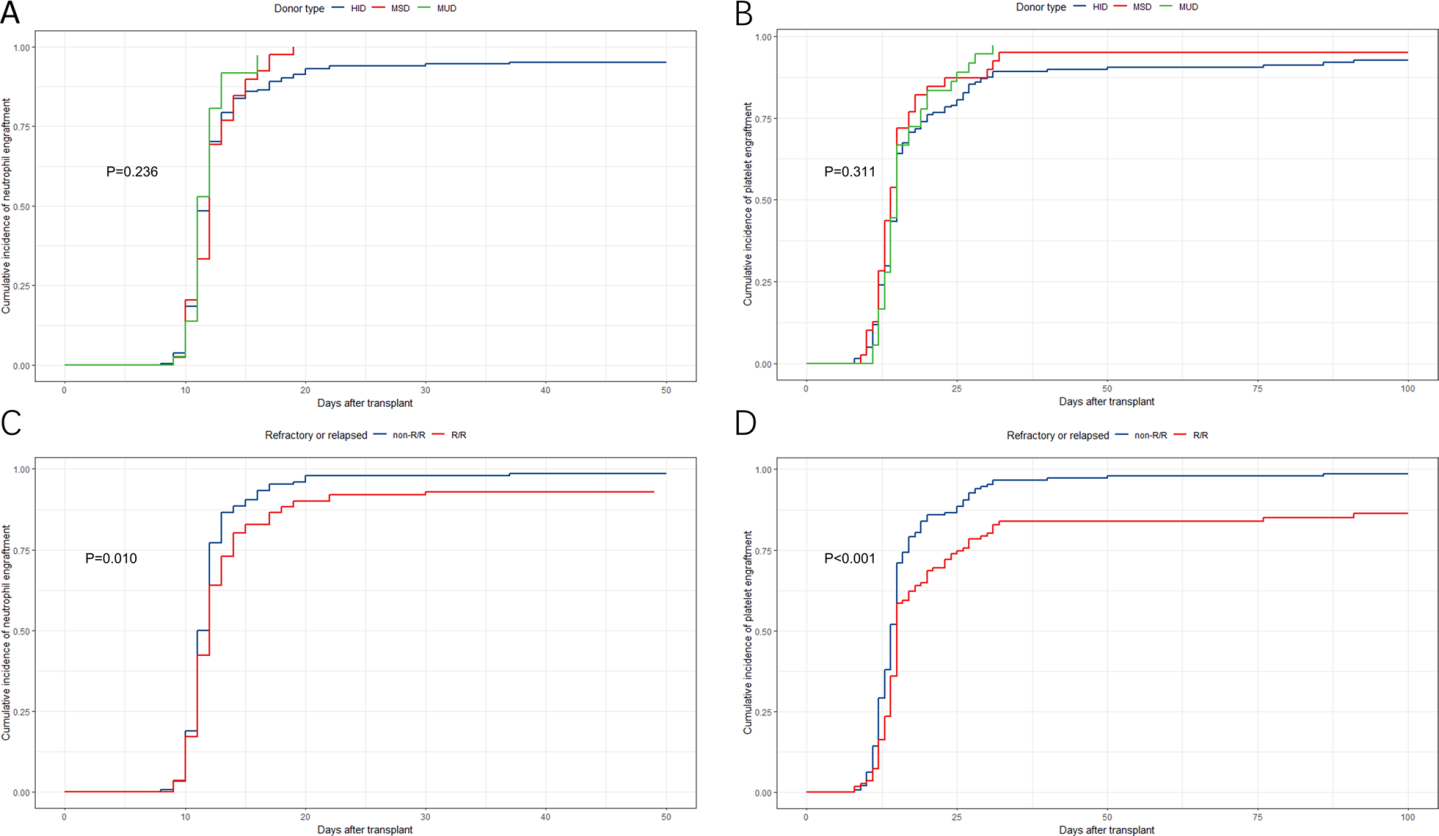
Outcomes of hematopoietic recovery. **A** Engraftment of neutrophil at 50d after allo-HSCT in different donor type cohort. **B** Engraftment of platelet at 100d after allo-HSCT in different donor type cohort. Engraftment of neutrophil (**C**) and platelet (**D**) between the refractory or relapsed (R/R) patients and the non-R/R

The median time for neutrophil recovery in the entire cohort was 12 days. The HID, MSD and MUD groups had median recovery times of 12 days (range: 8 to 37 days), 12 days (range: 9 to 19 days), and 11 days (range: 9 to 16 days), respectively, but there were no significant differences among these groups (P = 0.236) within 100 days. Additionally, there were no differences in the cumulative incidence of platelet engraftment among the three groups (15 days, range 8–91 days; 14 days, range 9–32 days; 15 days, range 11–31 days; P = 0.311) (Fig. 1A, B). However, patients with R/RAML/MDS had delayed neutrophil (P = 0.010, HR = 0.752) and platelet engraftment (P < 0.001, HR = 0.596) compared to the non-R/R group (Fig. 1C, D). Furthermore, older age (< 60 years) was associated with delayed neutrophil engraftment (P = 0.003, HR = 0.680 [0.525–0.881]), but not with platelet engraftment. High ECOG, high HCT-CI, and failure to reach CR1 before allo-HSCT were significantly associated with a delay in hematologic recovery.

### GVHD

**Fig. 2:**
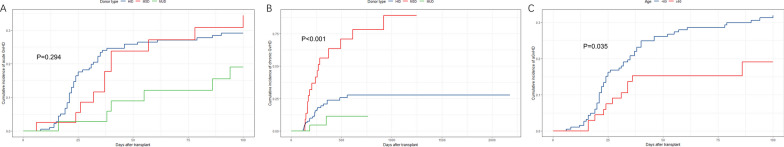
Graft-versus-host diseases after allo-HSCT. **A** Cumulative incidences of acute GvHD among different donor type cohorts. **B** Cumulative incidences of chronic GvHD in different donor type cohorts. **C** Cumulative incidences of acute GvHD between patients aged < 60y and ≥ 60y cohorts

The incidence of grade 2 to 4 acute GvHD 100 days after HSCT was 30% (23–37%) in HID, 35% (16–50%) in MSD, and 20% (4–33%) in MUD, with no significant differences found between the three groups (P = 0.294) (Fig. 2A). Additionally, no significant differences were observed among the groups when comparing the incidence of grade 3 to 4 acute GvHD. In a multivariable Cox regression model examining further factors associated with post-transplant acute GvHD, we found that younger patients (< 60 years) were independently associated with a higher risk of developing acute GvHD in the first 100 days (P = 0.035, HR = 1.942 [1.048–3.597] for grades II-IV acute GvHD) (Fig. 2C).

After adjusting for anti-HLA status, the 2-year cumulative incidences of chronic GvHD were 28% (17–37%) for HID, 78% (44–91%) for MSD, and 11% (0–25%) for MUD, with a significant difference, observed when comparing HID as the reference group (P < 0.001, HR = 4.391 [2.417–7.980] for MSD; P = 0.210, HR = 0.402 [0.096–1.677] for MUD) (Fig. 2B). Older age and diagnosis of refractory or relapsed diseases were not significant predictors of cumulative incidences of chronic GvHD. Further analysis revealed that the use of CD20 antibody or plasma exchange to target positive anti-HLA antibodies before transplantation was an independent factor preventing chronic GvHD (P = 0.014, HR = 0.126 [0.024–0.663]).

### Activation of CMV and EBV, with other complications

**Fig. 3:**
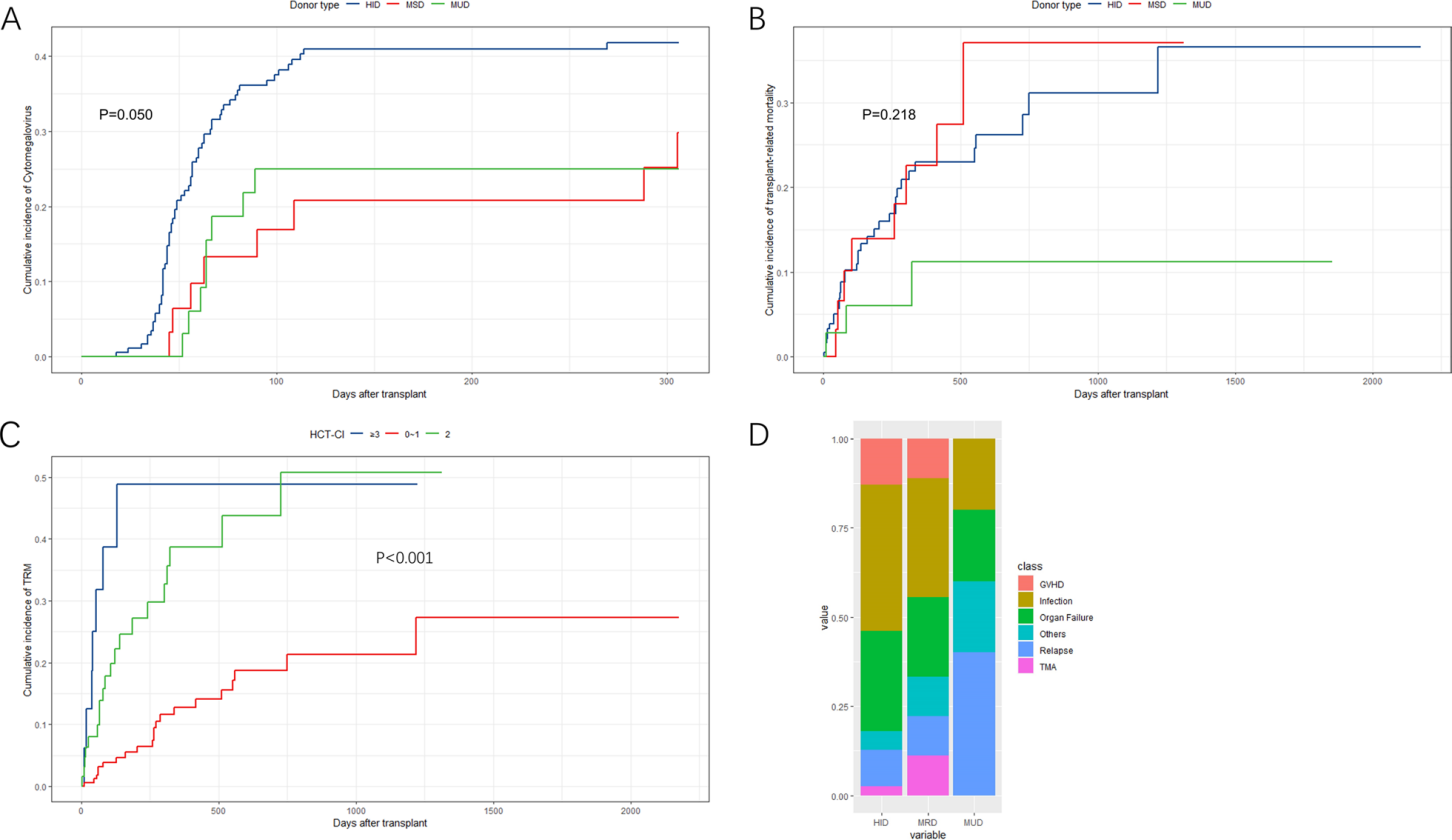
Transplant-related mortality, CMV infection and causes of death. **A** Cumulative incidences of Cytomegalovirus (CMV) infection in different donor type groups. **B** Cumulative incidences of transplant-related mortality in different donor type groups. **C** Cumulative incidences of transplant-related mortality in different levels of HCT-CI groups. **D** Causes of death in different donor type groups like severe GvHD, infection, organ failure, HSCT-associated thrombotic microangiopathy, relapse and so on (showing no significant difference)

The 1-year cumulative incidences of CMV reactivation were 45% (36–53%) for HID, 33% (10–50%) for MSD, and 26% (9–40%) for MUD, suggesting a potential increased risk associated with haploidentical transplantation compared to MUD (taking HIDs as reference: GP = 0.140, HR = 1.670 [MSD]; P = 0.038, HR = 2.110 [MUD]) (Fig. 3A). However, there was no difference observed in the occurrence of EBV reactivation among the three groups (P = 0.746). Additionally, comparable outcomes were observed between refractory and relapsed (R/R) and non-R/R groups, as well as between the two age groups divided by 60 years old.

During the follow-up period, three patients in the HID cohort were diagnosed with HSCT-associated thrombotic microangiopathy, while only one patient in the MSD group and none in the MUD group were diagnosed with this condition. Additionally, three HID patients, one MSD patient, and one MUD patient developed veno-occlusive disease. At 120 days post-transplant, the cumulative incidences of bloodstream infection were 8% (4–13%) in HID, 0% in MSD, and 4.7% (1–20%) in MUD, respectively, with no significant difference observed between the three groups (P = 0.856).

### TRM and mortality causes

Among the donor type groups, the 1-year probability of TRM was 23% (16–31%) in HID, 22% (8–40%) in MSD, and 11% (2–27%) in MUD. Our analysis did not show a significant difference in TRM when comparing MSD and MUD with HID (P = 0.240 for MSD, P = 0.660 for MUD, TRM) (Fig. 3B). The unadjusted promoting risk factors of TRM included older age, HCT-CI (greater than 2), and ECOG (greater than 3), while TCI (greater than 4) was linked with reduced TRM in the entire cohort. Considering the imbalanced potential multicollinearity, in the multivariate analysis of TRM, HCT-comorbidity index was the only independent risk factor (P < 0.001, HR = 2.336 [1.496–3.650]) in contrast to the limited importance provided by TCI (P = 0.110) (Fig. 3C) (Table 2). In a matched-pair analysis, there was no significant difference (P = 0.880) in TRM when comparing the Bu/Cy cohort with the Fludarabine-based cohort (Additional file 1: Fig. 1).

**Table 2:** Outcomes of transplantation according to donor type in multivariate analysis

Outcomes	Multivariate analysis	
	Hazard ratio (95%CI)	P-value
*Relapse*^†^		
HID	reference	
MSD	0.482 (0.158,1.473)	P = 0.200
MUD	0.654 (0.216,1.983)	P = 0.450
TRM*		
HID	reference	
MSD	1.207 (0.529,2.750)	P = 0.660
MUD	0.414 (0.121,1.420)	P = 0.160
*RFS*^‡^		
HID	reference	
MSD	0.747 (0.385,1.450)	P = 0.388
MUD	0.550 (0.247,1.224)	P = 0.143
*OS*^§^		
HID	reference	
MSD	0.863 (0.423,1.726)	P = 0.686
MUD	0.133 (0.192,1.243)	P = 0.133
GRFS*		
HID	reference	
MSD	1.609 (1.015,2.551)	P = 0.043*
MUD	0.471 (0.242,0.916)	P = 0.026*

^†^Relapse Adjusted for diagnosis (MDS: HR = 0.443, 95%CI, 0.208–0.946; P = 0.035), cytogenetic risk (Adverse risk: HR = 2.518, 95%CI, 1.361–4.658; P = 0.003), CR1 (Non-CR1: HR = 2.486, 95%CI, 1.307–4.729; P = 0.005)

^*^Transplant-related mortality (TRM) adjusted for age (over 60: HR = 1.548, 95%CI, 0.820–2.920; P = 0.180), HCT-CI (2 ~ 3: HR = 2.604, 95%CI, 1.301–5.210; P = 0.007. 4 ~ 6: HR = 5.060, 95%CI, 1.770–14.470; P = 0.002), TCI (over 4: HR = 0.598, 95%CI, 0.318–1.120; P = 0.110), refractory or relapsed disease status (R/R: HR = 1.582, 95%CI, 0.779–3.210; P = 0.200), and ABO blood type (Not matched: HR = 1.496, 95%CI, 0.813–2.750; P = 0.200)

^‡^Relapse-free survival (RFS) adjusted for HCT-CI (2 ~ 3: HR = 1.605, 95%CI, 0.951–2.712; P = 0.077. 4 ~ 6: HR = 3.609, 95%CI, 1.697–7.679; P < 0.001), TCI (Over 4: HR = 0.653, 95%CI, 0.406–1.048; P = 0.078), cytogenetic risk (Adverse risk: HR = 1.860, 95%CI, 1.161–2.979; P = 0.010), CR1 (Non-CR1: HR = 1.952, 95%CI, 1.194–3.191; P = 0.008)

^§^Overall survival (OS) adjusted for age (Over 60: HR = 1.762, 95%CI, 1.012–2.929; P = 0.043), diagnosis (MDS: HR = 1.722, 95%CI, 1.032–2.929; P = 0.045), HCT-CI (2 ~ 3: HR = 2.201, 95%CI, 1.235–3.923; P = 0.007. 4 ~ 6: HR = 3.225, 95%CI, 1.361–7.643; P = 0.008), cytogenetic risk (Adverse risk: HR = 1.603, 95%CI, 0.922–2.789; P = 0.095)

*GvHD, relapse-free survival (GRFS) adjusted for HCT-CI (2 ~ 3: HR = 1.786, 95%CI, 1.176–2.713; P = 0.007. 4 ~ 6: HR = 2.649, 95%CI, 1.421–4.937; P = 0.002), TCI (Over 4: HR = 0.663, 95%CI, 0.451–0.975; P = 0.036), CR1 (Non-CR1: HR = 1.952, 95%CI, 1.194–3.191; P = 0.008)

*Significant at P < 0.05

During the 2-year period following transplantation, a total of 39 patients in the HID cohort, nine patients in the MSD cohort, and five patients in the MUD cohort had died due to various causes, with no significant difference (P = 0.563) (Fig. 3D). Infection was the most common cause of death in both HID and MSD cohorts (41% and 33%, respectively). Meanwhile, none of the patients in the MUD cohort had died of GvHD or thrombotic microangiopathy in the 2-year follow-up.

### Relapse, RFS, OS, and GRFS

**Fig. 4:**
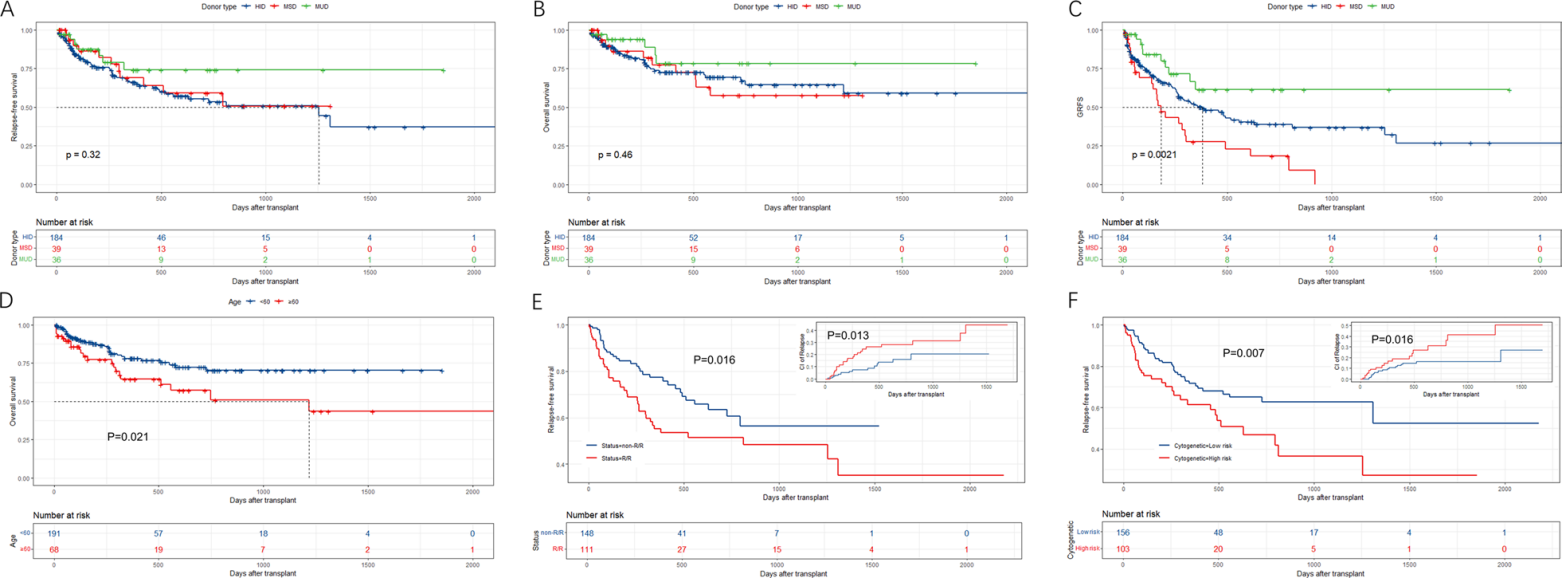
Prognostic outcomes of transplantation. **A** Relapse-free survival (RFS) in different donor type groups. **B** Overall survival in different donor type groups. **C** GvHD, Relapse-free survival in different donor type groups. **D** Overall survival in the aged < 60y and ≥ 60y cohorts. **E** RFS and cumulative incidence of relapse across the R/R and non-R/R cohort. **F** RFS and cumulative incidence of relapse across the low cytogenetic risk and higher cytogenetic risk cohort

Comparing the three cohorts, there were similar outcomes for relapse and RFS, whether using univariate or multivariate analysis (Fig. 4A, C). Refractory or relapsed disease status and adverse cytogenetic risk were associated with higher relapse rates and inferior RFS. (P = 0.013, P = 0.016, respectively, for relapse; P = 0.016, P = 0.007, respectively, for RFS) (Fig. 4E, F). The unadjusted 2-year OS was 67% (58–77%) in the HID cohort, 58% (40–82%) in MSD, and 78% (63–98%) in MUD, but the difference was not statistically significant (P = 0.460) (Fig. 4B).

In univariate analysis, older age (over 60) with high HCT-CI was associated with decreased OS (Fig. 4D) and RFS (P = 0.021, P < 0.001, for OS; P = 0.048, P < 0.001, for RFS). Risk factors associated with reduced GRFS included ECOG (P = 0.012, HR = 1.560 [1.104–2.203]), adverse cytogenetic risk (P = 0.003, HR = 1.730 [1.202–2.490]), HCT-CI (P < 0.001, HR = 1.887 [1.451–2.455]), TCI (P = 0.005, HR = 0.582 [0.401–0.845]), and MSD donor type. Compared to HID, MSD had inferior GRFS (P = 0.036, HR = 1.630), while MUD had better survival (P = 0.026, HR = 0.471) when accounting for cytogenetic risk, HCT-CI, and TCI (Table 2). When considering chronic GVHD as a time-dependent covariate for OS, we found that its occurrence independently led to a deterioration in overall survival (P = 0.006, HR = 2.971) after adjusting for age, diagnosis, and HCT-CI. In addition, in order to alleviate the potential disturbance from the different numbers of samples in the donor type cohorts, we applied a matched-pair analysis and received the similar consequences as the former in the multivariable analysis (Additional file 1: Table 1).

Of note, when all the patients were divided into the AML and MDS cohorts according to diagnosis, we found patients in the MDS cohort were associated with higher risk for inferior overall survival (P = 0.045, HR = 1.722) (Additional file 1: Fig. 2A) but with lower relapse rate (P = 0.035, HR = 0.443) (Additional file 1: Fig. 2B) in a multivariate analysis. There was a higher incidence of extensive chronic GvHD (P = 0.004, HR = 0.193) (Additional file 1: Fig. 2C) in the MDS cohort than in the AML cohort. In this way, patients in the MDS cohort were more likely to die from severe GvHD (P = 0.046, HR = 0.114) (Additional file 1: Fig. 2D). While in a deeper analysis involved with donor type, the choice of donor type exerted no significant influence on the outcomes like relapse, transplant-related mortality, relapse-free survival and overall survival in the AML and MDS cohort respectively (Additional file 1: Table 2). In the AML cohort, MSD became a high risk for lower GRFS (P = 0.012, HR = 2.140) compared with HID while such situation was not obvious in the MDS cohort.

## Discussion

In this retrospective study, we analyzed data from a single center from patients aged over 55 years who underwent transplantation using HID, MSD, and MUD, respectively. As a real-world study, we aimed to balance the deviations caused by excluding infeasible patients while acknowledging the bias from basic characteristics due to expanded access [19]. We included 111 (42.86%) refractory or relapsed aged patients and used multivariate analysis to mitigate the inherent bias. Our findings indicate no significant differences in TRM, RFS, incidence of relapse, and OS among the HID, MSD, and MUD groups. However, we observed a significantly inferior outcome of GRFS in the MSD cohort compared to that of HID and MUD.

Our results indicated that compared with HID, MSD, and MUD presented comparable outcomes of hematopoietic recovery. However, relatively lower engraftment in the HID cohort showed the potential risk factor for the HLA disparity. Considering the threatening relationship of anti-HLA antibodies with graft failure [20], strategies before allo-HSCT in our studies made an effective improvement in balancing the delay of engraftment. In line with the previous study [21], the status of the diseases before allo-HSCT brought about a negative influence on hematopoietic recovery. More actions are in unmet need for improving the post-transplant situations of those patients with advanced disease status.

The MSD cohort showed a higher incidence of extensive chronic GvHD compared to the better circumstances in the HID cohort. This may be attributed to PT-Cy GvHD prophylaxis applied in HID [22, 23]. Furthermore, a higher proportion of peripheral blood graft sources in the MSD cohort may be responsible for a higher incidence of limited or extensive chronic GvHD [24]. Unfortunately, we were able to compare interventions among GvHD prophylaxis, graft source, and the donor type in this study. Consistent with a previous systematic review and meta-analysis, a reduction in chronic GvHD did not result in more relapse [25]. However, in our study, infection-related mortality was found to be higher in HID. It is worth noting that plasmapheresis or rituximab administrated before HSCT to remove in-vivo positive HLA-antibodies could significantly prevent the occurrence of limited or extensive chronic GvHD. This is an interesting finding, as a previous study has correlated the function of B cells in chronic GvHD [26]. However, further research is required to reach a consensus on this matter. Regarding acute GvHD, age above 60 years was found to be an independent protective factor for the incidence of acute GvHD, and this effect remained after paired matching. However, its existence in younger adults is scarcely reported [27], leaving a gap in the research for older adults.

Based on our analysis, HID showed comparable TRM, incidence of relapse, RFS, and overall survival to MSD and MUD, suggesting that HID could be a feasible transplantation option for patients lacking qualified MSD or MUD donors. Previous studies have also confirmed the equivalence of donor type in prognosis for allo-HSCT [4–10]. Additionally, the age-limited threshold of 60 has become blurred and indistinct [28]. Thus, high-profile patients should consider transplant-related mortality and overall survival, which are linked to higher HCT-CI score, as highlighted by our multivariate analysis and dozens of previous studies [29]. This underscores the importance of assessing comorbidity before HSCT. Zero fatal case due to GvHD and HSCT-associated thrombotic microangiopathy in the MUD cohort was seen in the follow-up though presenting no significant importance with limited examples, which might indicate the more solid safety and better life after MUD transplantation. Our study found that failure to reach CR1 and high cytogenetic risk were associated with significantly shorter RFS. Patients with R/R AML/MDS tended to relapse more frequently and may have dismal overall survival. HSCT has the potential to prolong overall survival or even cure leukemia patients. A previous report showed similar 2-year incidence of relapse (29% vs. 28% in our study) and 2-year RFS (51% vs. 51% in our study) in patients with R/R disease status. Most of these patients had satisfactory post-transplantation outcomes in terms of quality of life, and the selection of donor type did not have a significant impact on their prognosis [30]. In the past two decades, the use of hypomethylating agents and targeted therapies before transplantation has improved the outcomes of allo-HSCT in refractory or relapsed patients [31]. However, primary resistance to induction regimens or relapses after conditioning treatment can lead to delayed engraftment and dismal outcomes in allo-HSCT, resulting from advanced diseases or positive minimal residual diseases. Therefore, patients with R/R diseases require priority attention. Inferior overall survival in patients with MDS than those with AML also provoked our attention in our research and was finally considered to be relevant with higher occurrence of GvHD-related mortality which resulted from more frequent extensive chronic GvHD in the MDS patients.

GRFS is a sensitive endpoint for assessing the quality of life and health status of post-HSCT individuals [11], particularly for older adults. With the fine distinction of GRFS in MUD from the American study enrolling patients aged ≥ 60 years old [4], our study presented better adjusted GRFS in the MUDs. Considering the similar outcomes between post-transplantation cyclophosphamide (PT-Cy) and conventional prophylaxis with ATG [32], donor age could be the potential factor leading to the different outcomes in the two studies. In addition, high intensity regimen (Bu/Cy) before HSCT independently prolonged GRFS in elderly patients in comparison with the fludarabine-based regimen partly by circumventing the rejection from residual host cells [33], since the high intensity regimen was not significantly expected to give rise to a higher transplant-related mortality in our study.

As a previous report indicated, advanced donor age in patients over 40 years was correlated with increased TRM and inferior leukemia-free survival translating into lowered OS [34]. Patients younger than 55 years tend to have a choice of a haploidentical donor from their offspring or an HLA-matched donor from their sibling relatives. In contrast, those older than 55 years tend to be limited to receiving grafts from a haploidentical donor among their offspring in China. A multi-center study in China favoured the significant advantage of survival outcomes in the HIDs over 50 years from young offspring donors compared to the MSDs from their sibling donors [35]. Another study enrolling 1082 patients aged from 55 to 76 years old showed a lower incidence of chronic GvHD but higher non-relapse mortality in offspring donors compared to HLA-matched sibling donors [22]. A retrospective analysis including 406 older patients who underwent allo-HSCT demonstrated that HIDs managed to achieve similar survival and significantly lower rates of chronic graft-versus-host compared with MSD [8]. Contrary to our study, MUD from all age ranges (from 35 to 54 years) was featured with a significantly inferior curve of GRFS compared to HID. Therefore, when considering our relatively high proportion of patients diagnosed with refractory or relapsed AML/MDS, MUDs from younger donors in our study presented better adjusted GRFS with lower overall occurrence of poor events resulting in decay in the quality of life for aged patients. Another study related to donor age in MUDs determined that every 10-year increment in donor age led to a 5.5% increase in the HR for overall mortality [11]. Overall, better performance in MUDs was likely due to relatively younger donors as stated in a previous review that cumulative incidences of indeterminate mutations or impairments in blood stem cells with increasing age have the potential to impact clonal expansion or even denote a malignancy [36]. In our study, we have demonstrated the significant advantage of MUD with younger donors over MSD in the quality of life for elderly patients through the way of employing a limited population. What is more, we have got similar prognosis to the previous studies in HID with MUD after expanding HIDs to larger populations. Due to the special situations mentioned above, we were unable to take more MSDs into considerations. So further prospective studies involving this approach are necessary.

There were limitations in our analysis. In addition to the retrospective design and inherent selection bias, the type of GvHD prophylaxis was linked to the choice of donor, making it difficult to evaluate its impact on allo-HSCT outcomes. Moreover, there are limited studies on the donor age of haploidentical sibling donors, MSD, and MUD in patients aged 55 years or older with AML or MDS. Therefore, we should expand our study population to provide guidance on the best donor choice for elderly patients.

Despite its limitations, our study confirms the feasibility of using HID as a viable option for transplantation patients without access to qualified MSD or MUD. However, post-transplant infections should be closely monitored. Considering the better-adjusted GRFS in MUD from younger donors, MUD appears to be a better choice for elderly patients compared to MSD, especially for those with R/R AML/MDS. For those in need of urgent transplantation, HID is a valid alternative at present.

## Conclusion

This retrospective study presented the clinical feasibility of HID transplantation in elderly patients with AML/MDS, in comparison with MSD and MSD. This outcome may help to alleviate the shortage of donor for allo-HSCT in elderly patients. What is more, based on the predominant situation of younger donors in HID and MUD in China, we found that younger donors had brought a better quality of life (GRFS) to elderly patients in post-transplant follow-up. And a more detailed and large-scale prospective study needs to be conducted to consolidate this result.

### Supplementary Information

**Additional file 1.** Supplementary Figure S1, S2 and Table S1, S2.

## Data Availability

The datasets used during the current study are available from the corresponding author on reasonable request.

## Abbreviations

AML: Acute myeloid leukemia
MDS: Myelodysplastic syndrome
Allo-HSCT: Allogenic hematopoietic stem cell transplantation
MSD: Matched sibling donor
MUD: Matched unrelated donor
HID: Haploidentical identical donor
OS: Overall survival
RFS: Relapse-free survival
R/R: Refractory or relapsed
ECOG: Eastern Cooperative Oncology Group Zubrod performance status
HCT-CI: The hematopoietic cell transplantation comorbidity index
CR1: The first complete remission
TCI: Transplant conditioning intensity score
TRM: Transplant-related mortality
GRFS: Graft-versus-host-disease-free, relapse-free survival
GvHD: Graft-versus-host disease
CMV: Cytomegalovirus
EBV: Epstein–Barr virus
Bu/Cy: Busulfan and Cyclophosphamide
