# Supplementary material for: Allogenic hematopoietic stem cell transplantation outcomes of patients aged ≥ 55 years with acute myeloid leukemia or myelodysplastic syndromes in China: a retrospective study
Source: Stem Cell Res Ther. 2024 Jan 29;15:24. doi: 10.1186/s13287-024-03640-4 (PMC10823660; doi:10.1186/s13287-024-03640-4)
Supplement: Supplementary file 1 — Additional file 1. Supplementary Figure S1, S2 and Table S1, S2. [file 13287_2024_3640_MOESM1_ESM.docx]

**Supplementary materials include Fig. 1S to 2S and Table 1S to 2S.**

**Fig. 1S** Propensity score matching was applied to evaluate the effects of the two different regimens on the outcomes of allo-HSCT. We got 60 patients respectively in the two cohorts. The figure listed below showed the Kaplan-Meier plot of transplant-related mortality in the matched-pair Bu/Cy regimen and Flu-based regimen cohorts.


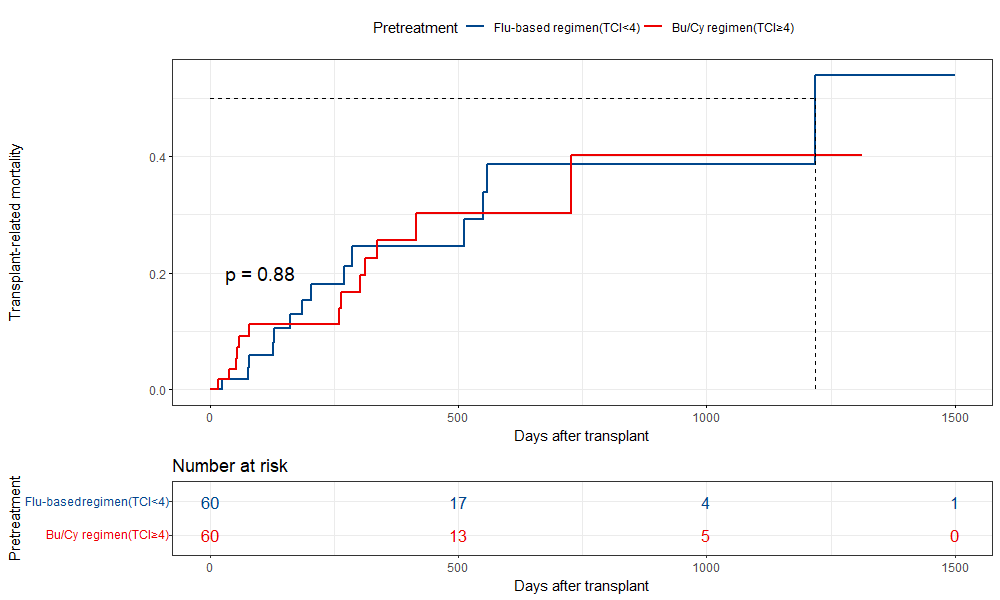


**Supplementary figure 1. Transplant-related mortality in the Bu/Cy regimen and Flu-based regimen cohort.** Transplant-related mortality for propensity score-matched analysis of patients receiving the Flu-based regimen (N=60) versus patients receiving the Bu/Cy regimen (N=60).

**Fig. S2** We also did an analysis of transplant-related outcomes in the AML and MDS cohort. These significant results showed GvHD-related effects on the situations of overall survival and relapse in the AML and MDS cohorts.


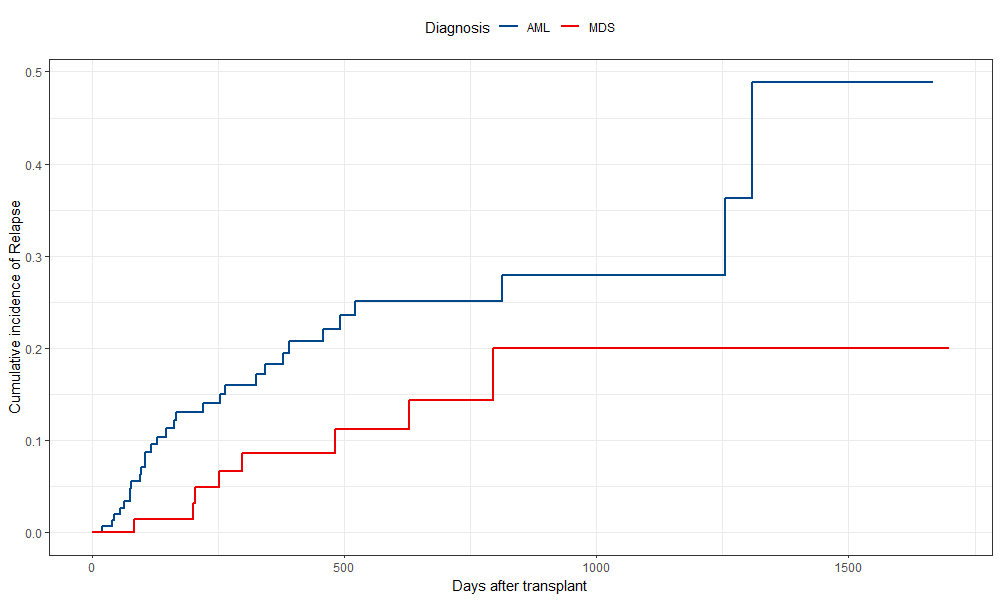

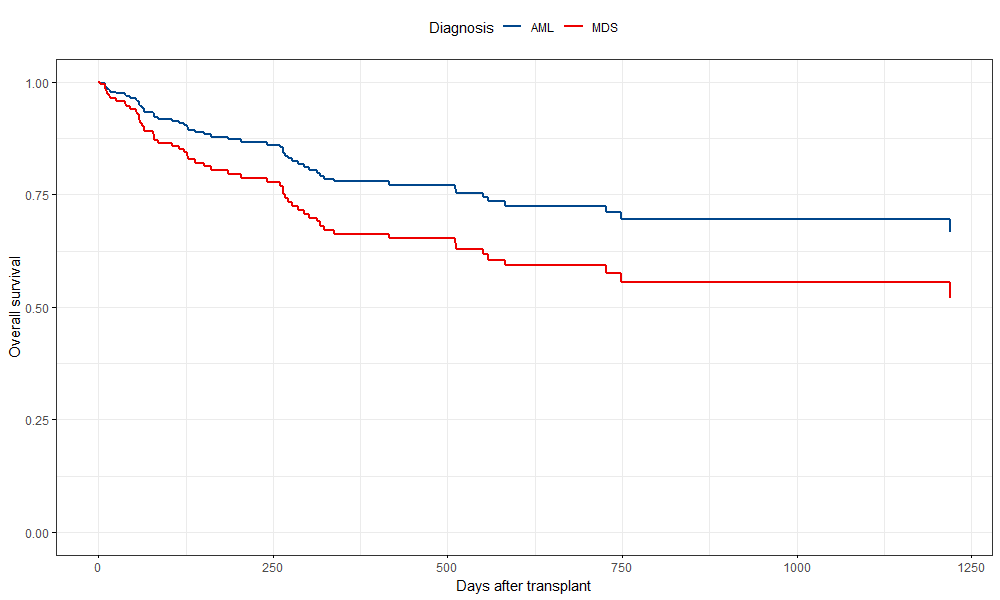

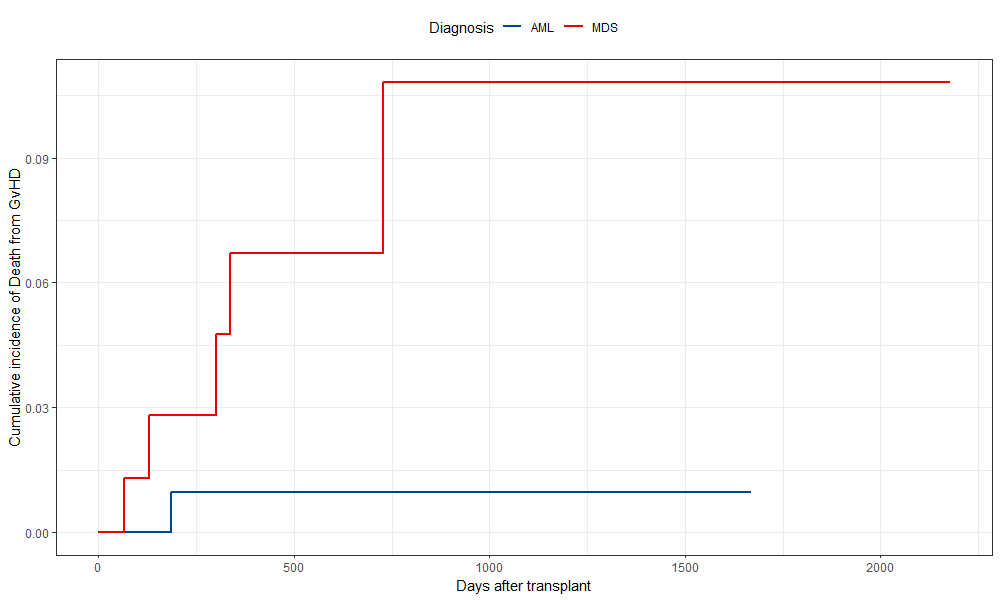


A

P=0.045

B

P=0.035


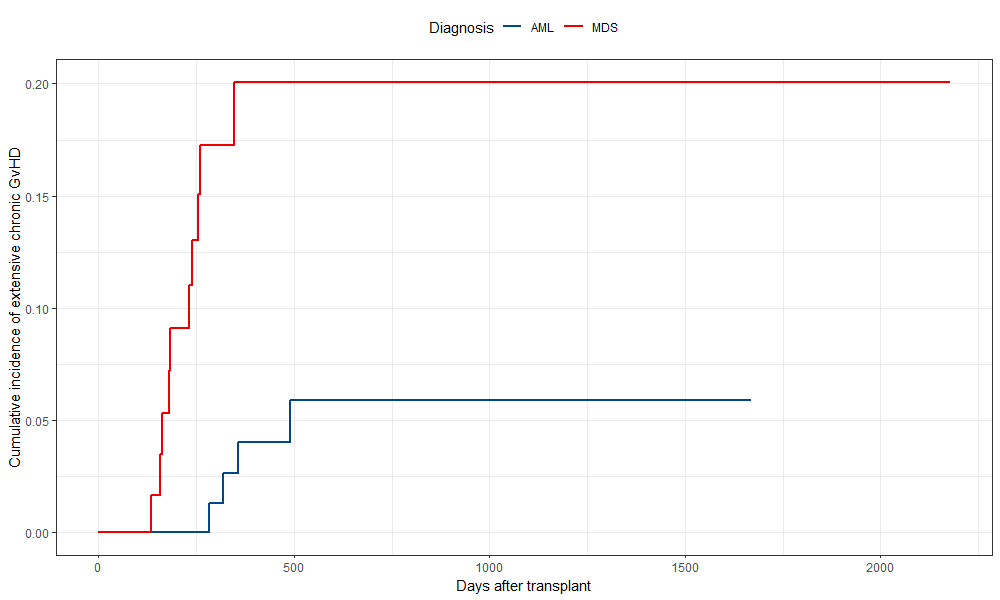


C

P=0.004

D

P=0.046

**Supplemental figure 2. Outcomes of allo-HSCT in the AML and MDS cohorts.** Adjusted overall survival (adjusted for donor type, age, HCT-CI and cytogenetic risk) (A), relapse (B) in the AML and MDS cohorts. (C) Cumulative incidence of extensive chronic graft-versus-host disease in the AML and MDS cohorts. (D) Cumulative incidence of graft-versus-host disease-related mortality in the AML and MDS cohorts.

**Table S1.** Taking the significant difference in the respective samples in the donor type cohorts into consideration, we also applied propensity matching in the analysis of the donor type cohorts

**Supplementary Table 1. Outcomes of HSCT in the different donor type cohorts with matched-pair analysis (propensity score matching).**

| **Variants (HID as reference)** | **HID (n=36) versus MSD (n=36)** | | | |  | | **HID (n=31) versus MUD (n=31)** | | | |
| --- | --- | --- | --- | --- | --- | --- | --- | --- | --- | --- |
|  | **Hazard ratio (95%CI)** | | **P-value** |  | | **Hazard Ratio (95%CI)** | | | **P-value** |  |
| Relapse | 0.817 (0.186,3.580) | P=0.790 | | |  | | 0.759 (0.369,1.560) | P=0.450 | | |
| Transplant-related mortality | 2.460 (0.645,9.360) | P=0.190 | | |  | | 0.888 (0.434,1.810) | P=0.740 | | |
| Relapse-free survival | 1.523 (0.547,4.240) | P=0.421 | | |  | | 0.633 (0.225,1.782) | P=0.386 | | |
| Overall survival | 2.002 (0.611,6.559) | P=0.252 | | |  | | 0.643 (0.181,2.284) | P=0.495 | | |
| GvHD, relapse-free survival | 3.230 (1.538,6.782) | P=0.002* | | |  | | 0.565 (0.234,1.367) | P=0.205 | | |

***Significant at P<0.05**

**Table S2.** Considering the influences of different diseases in patients on the outcomes of allo-HSCT, we analyzed the outcomes of allo-HSCT from donor type in the AML and MDS cohorts.

| **Variants** | **AML** | | | |  | | **MDS** | | | |
| --- | --- | --- | --- | --- | --- | --- | --- | --- | --- | --- |
|  | **Hazard ratio (95%CI)** | | **P-value** | |  | | **Hazard Ratio (95%CI)** | | **P-value** | |
| Relapse |  | |  | |  | |  | |  | |
| HID | 1.000 | |  | |  | | 1.000 | |  | |
| MSD | - | | - | |  | | 2.060 (0.491,8.670) | | P=0.320 | |
| MUD | 0.679 (0.199,2.320) | | P=0.540 | |  | | 1.030 (0.118,9.060) | | P=0.980 | |
| Transplant-related mortality |  | |  | |  | |  | |  | |
| HID | 1.000 | |  | |  | | 1.000 | |  | |
| MSD | 1.282 (0.428,3.840) | | P=0.660 | |  | | 0.815 (0.329,2.020) | | P=0.660 | |
| MUD | 0.595 (0.140,2.530) | | P=0.480 | |  | | 0.194 (0.025,1.500) | | P=0.120 | |
| Relapse-free survival |  | |  | |  | |  | |  | |
| HID | 1.000 | |  | |  | | 1.000 | |  | |
| MSD | 0.650 (0.232,1.825) | | P=0.414 | |  | | 0.859 (0.364,2.029) | | P=0.729 | |
| MUD | 0.790 (0.310,2.012) | | P=0.622 | |  | | 0.289 (0.067,1.234) | | P=0.094 | |
| Overall survival |  | |  | |  | |  | |  | |
| HID | 1.000 | |  | |  | | 1.000 | |  | |
| MSD | 1.099 (0.380,3.184) | | P=0.861 | |  | | 0.834 (0.330,2.112) | | P=0.703 | |
| MUD | 0.768 (0.230,2.559) | | P=0.667 | |  | | 0.347 (0.080,1.496) | | P=0.156 | |
| GvHD, relapse-free survival |  | |  | |  | |  | |  | |
| HID | 1.000 |  | |  | | 1.000 | |  | |  |
| MSD | 2.140 (1.184,3.868) | P=0.012* | |  | | 1.233 (0.616,2.465) | | P=0.554 | |  |
| MUD | 0.511 (0.204,1.281) | P=0.152 | |  | | 0.511 (0.198,1.318) | | P=0.165 | |  |

**Supplementary Table 2. Outcomes of allo-HSCT in the AML and MDS cohorts with a univariate analysis.**

***Significant at P<0.05.**
